# Imaging and Management of Incidental Renal Lesions

**DOI:** 10.1155/2017/1854027

**Published:** 2017-05-31

**Authors:** Silvio Mazziotti, Giuseppe Cicero, Tommaso D'Angelo, Maria Adele Marino, Carmela Visalli, Ignazio Salamone, Giorgio Ascenti, Alfredo Blandino

**Affiliations:** Department of Biomedical Sciences and Morphological and Functional Imaging, University of Messina, Policlinico “G. Martino”, Via Consolare Valeria 1, 98100 Messina, Italy

## Abstract

The increased use of imaging modalities in the last years has led to a greater incidence in depicting abdominal incidental lesions. In particular, “incidentalomas” of the kidney are discovered in asymptomatic patients or patients who suffer from diseases not directly related to the kidneys. The aim of this paper is to provide the radiologist with a useful guide to recognize and classify the main incidental renal findings with the purpose of establishing the correct management. First we describe the so-called “pseudotumors” which are important to recognize in order to avoid a misdiagnosis. Afterwards we categorize true renal lesions into cystic and solid types, reporting radiological signs helpful in differentiating between benign and malignant nature.

## 1. Introduction

The majority of renal masses are found incidentally as a result of the widespread use of ultrasonography (US) and computed tomography (CT) as well as magnetic resonance imaging (MRI) performed for problems often unrelated to the kidneys. Furthermore, technological improvements have increased the spatial, contrast, and temporal resolution of these imaging modalities allowing for higher rates of detection.

Therefore, so often, these incidental renal masses are recognized in patients without symptoms directly ascribable to the kidneys.

In an aging population, the incidence of renal incidentalomas is rising because the prevalence of both renal cysts and renal cell carcinoma (RCC) increases with the age. Autopsy results have shown that almost half of people older than 50 years have one or more renal masses. Most of these represent simple cysts that can be easily diagnosed as benign on the basis of imaging and do not require treatment. However, complex cystic and solid renal masses are also discovered, many of which are clearly malignant and need to be surgically removed, while others may not require surgical intervention.

In any case, despite the most frequent benign nature of the incidental renal lesions, their discovery often produces a cascade of costly examinations also determining patient's anxiety and unnecessary radiation exposure.

In this context, whenever an incidental renal mass is found, it is important (a) to establish the most likely diagnosis on the basis of imaging findings and (b) to set the correct management for possible malignant lesions (e.g., close follow-up, change imaging technique, percutaneous biopsy, surgery, or ablation).

The aim of this paper is to provide the radiologist with a useful guide for the most correct interpretation of incidental renal findings in order to distinguish surgical from nonsurgical lesions.

Initially, we describe pseudotumors, a common pitfall in the radiological approach to renal incidentalomas. Afterwards, we categorize true renal lesions into cystic and solid types, reporting radiological signs helpful in differentiating their behavior, from benign to potentially serious, including malignant.

## 2. Pseudotumors

“*Pseudotumors*” are common findings which can mimic a mass for their appearance; the most common ones are the hypertrophied column of Bertin and the lobar dimorphisms (like the “dromedary hump” or the persistent fetal lobulations).

**Figure 1:**
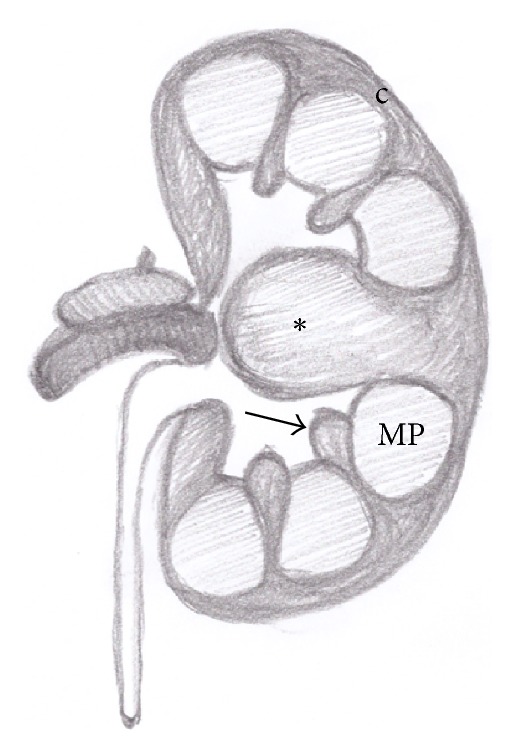
Anatomical drawing of a hypertrophied column of Bertin (asterisk). Normal renal column (arrow); medullary pyramid (MP); renal cortex (c).

The* hypertrophied column of Bertin* (also known as “septa of Bertin”) is a common anatomical variant consisting in a “mass-like” enlargement of the cortical tissue normally present between the renal pyramids. It is usually located in the middle third of the kidney, more commonly on the left side (Figure 1) [1].

**Figure 2:**
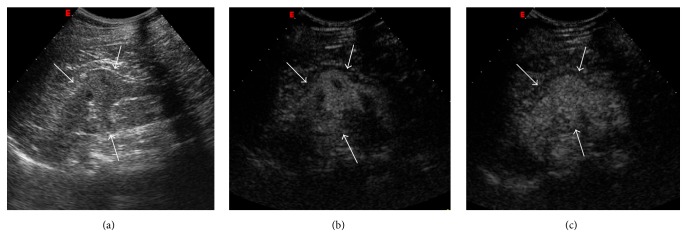
Hypertrophied column of Bertin. Gray scale US (a); CEUS (b, c). Gray scale US shows a “mass-mimicking” unfolding of cortical renal tissue (arrows) between renal medullary pyramids. After contrast medium administration, the enhancement is similar to the surrounding cortical parenchyma.

Sonography can easily recognize this condition, characterized by the same echogenicity of renal parenchyma, smooth renal contour, and lack of acoustic posterior enhancement (Figure 2(a)) [2]. Moreover, on Doppler US, septa of Bertin show arterial and venous flow pattern similar to the renal parenchyma [3].

Contrast-enhanced US (CEUS) can be a useful tool to confirm the normal cortical tissue interposed between medullary pyramids (Figures 2(b) and 2(c)).

Sometimes an atypical appearance may require further evaluation with cross-sectional imaging. Hypertrophic column of Bertin is isodense at CT and isointense at MRI to the normal renal parenchyma.

**Figure 3:**
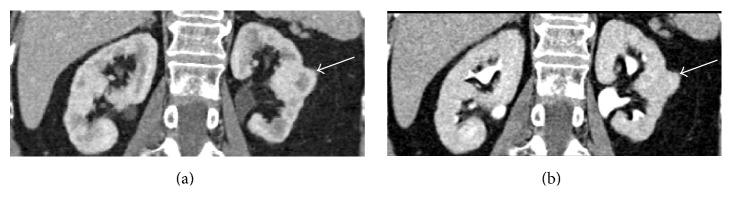
Hypertrophied column of Bertin. Coronal reformatted contrast-enhanced CT images in corticomedullary (a) and nephrographic phase (b) well demonstrate the enlarged column of Bertin (arrows) characterized by the same pattern of enhancement of the normal renal cortex.

**Figure 4:**
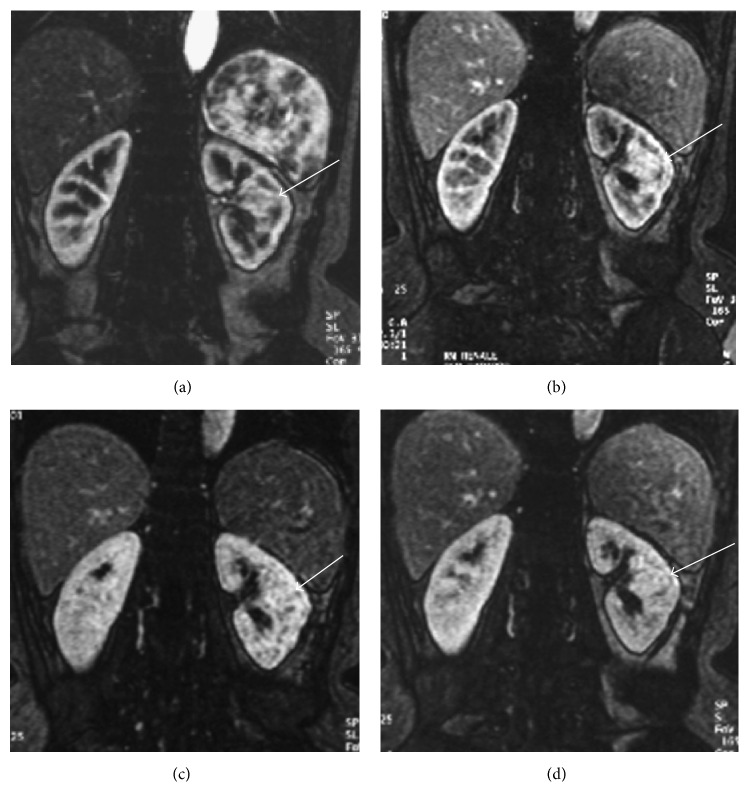
Hypertrophied column of Bertin. Coronal GE T1-weighted fat-sat gadolinium enhanced images (a–d) showing a mass-like finding in the left kidney (arrow). The prominent column of Bertin is in continuity with the renal cortex and manifests the same enhancement of the renal parenchyma in all contrastographic phases.

**Figure 5:**
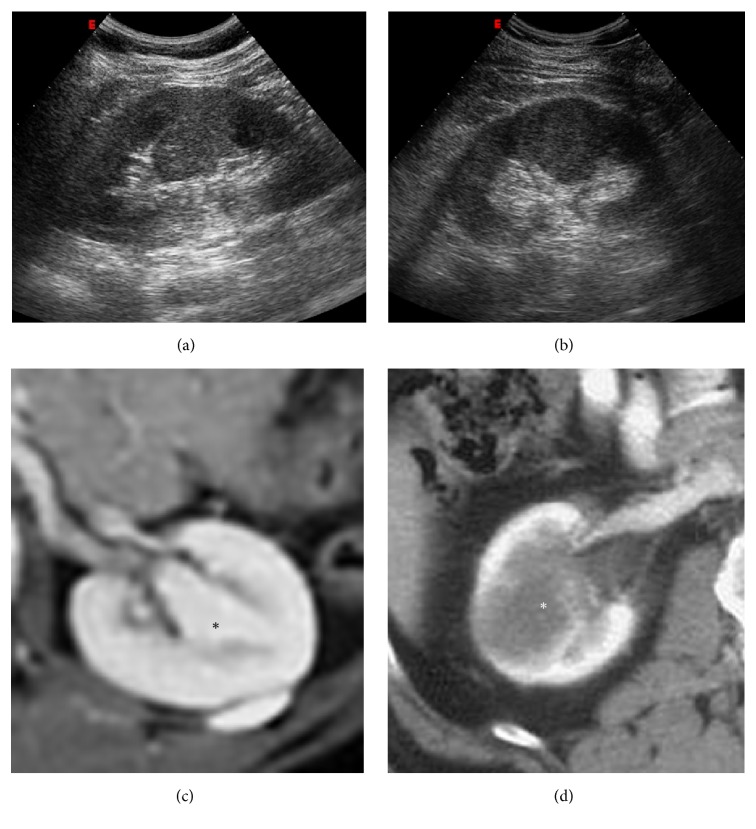
Pseudotumor versus true solid renal lesion. Gray scale US (a, b) and contrast-enhanced CT (c, d). Renal incidentalomas in two different patients with similar echogenicity pattern (a, b). Contrast-enhanced CT reveals a hypertrophied column of Bertin in the first case (black asterisk in (c)) and a solid hypovascular mass in the second case (white asterisk in (d)).

Contrast-enhanced imaging (US, CT, and MR) will show the same enhancement pattern of the surrounding renal parenchyma (Figures 3(a), 3(b), and 4) allowing the differential diagnosis between pseudotumors and infiltrative solid renal lesion (Figure 5) [2, 4, 5].

*Dromedary hump* or* splenic hump* appears as a focal bulge on the lateral border of the left kidney, caused by the splenic impression on its superolateral contour. It can be easily diagnosed with sonography due to the same echogenicity of the renal parenchyma and normal blood flow at color Doppler and CEUS [4, 5]. At unenhanced and contrast-enhanced CT and MRI it shows the same features of the normal renal parenchyma.

**Figure 6:**
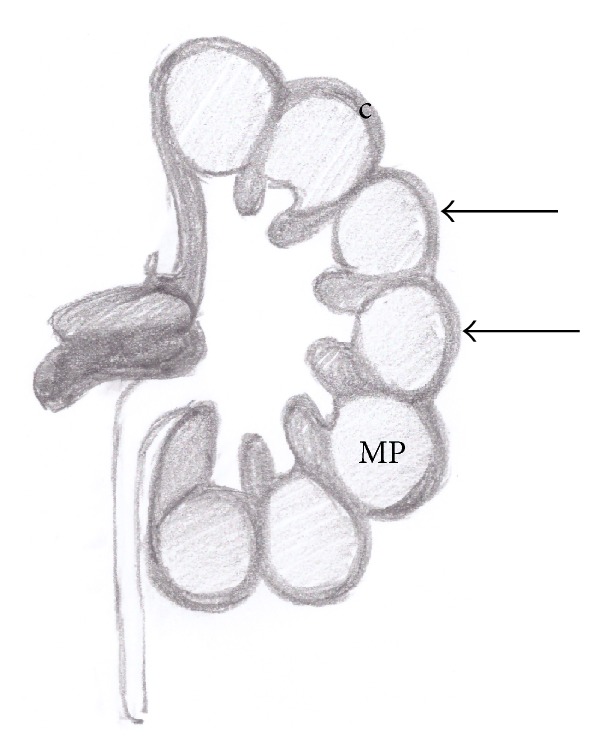
Anatomical drawing of persistent fetal lobulations (arrows). Medullary pyramid (MP); renal cortex (c).

**Figure 7:**
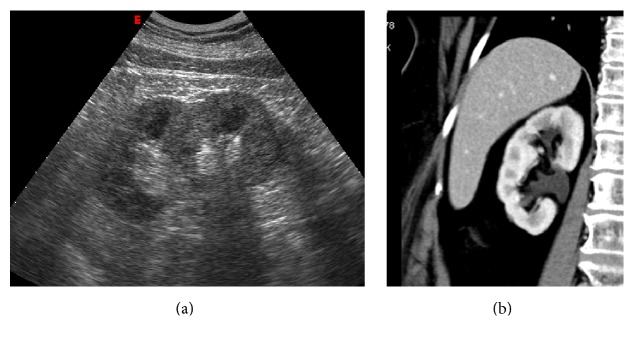
Persistent fetal lobulations. US image (a) and multiplanar coronal contrast-enhanced CT obtained in corticomedullary phase (b). The typical appearance of the normal congenital variant is depicted on both US and contrast-enhanced CT.

*Persistent fetal lobulation (lobation)* is a normal variant diagnosed in 4% of children and 10% of adult population. It consists of an indentation of the renal surface in between the renal pyramids, caused by an incomplete fusion of the renal lobules during early childhood (Figures 6, 7(a), and 7(b)) [5, 6]. When depicted in adult kidneys, it can be misdiagnosed with a tumor or a renal scar.

**Figure 8:**
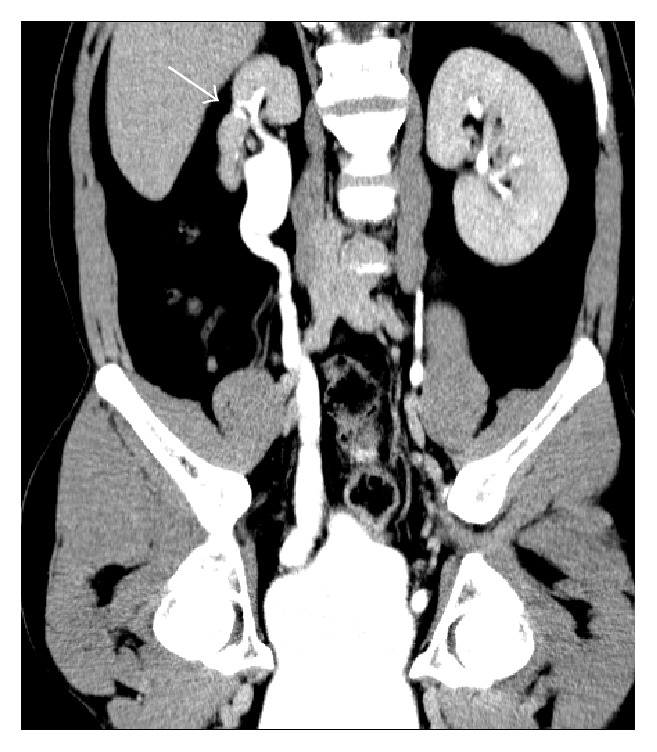
Postpyelonephritic scar. Coronal reformatted contrast-enhanced CT (excretory phase) shows a focal postpyelonephritic scar (arrow) in the upper-third of the right kidney with dilatation of ipsilateral renal pelvis and ureter.

Postpyelonephritic renal scars can be easily distinguished because they usually overlay the medullary pyramids with calyceal clubbing due to the retraction of the papilla from the scar (Figure 8).

*Infectious processes* (pyelonephritis, abscesses) or* traumatic injuries* were excluded from this topic because of the symptoms and the clinical history of the patient.

## 3. Cystic Lesions

The majority of cystic lesions incidentally discovered at imaging are simple cysts which are easily diagnosed and do not require further follow-up or treatment. However, complex cysts that need a more careful evaluation are not so rare [7].

The Bosniak classification is an evaluating system of cystic renal masses, originally based only on contrast-enhanced CT findings but then commonly applied to US and MRI. It is used to categorize a cystic renal mass according to the risk of malignancy into one of five categories (I, II, IIF, III, and IV) and to suggest the consequent follow-up or treatment [7, 8].

In particular, a correct Bosniak classification of a cystic renal lesion requires the i.v. administration of contrast medium, in order to evaluate the enhancement of septa, walls, or nodules.

In this regard contrast-enhanced multiphasic evaluation at cross-sectional imaging, composed of corticomedullary, early and delayed nephrographic phases, is usually performed.

A further help in depicting even small amounts of intralesional contrast medium enhancement can also come from CEUS, dual-energy CT, and dynamic contrast-enhanced subtraction MRI [7, 9–12].

**Figure 9:**
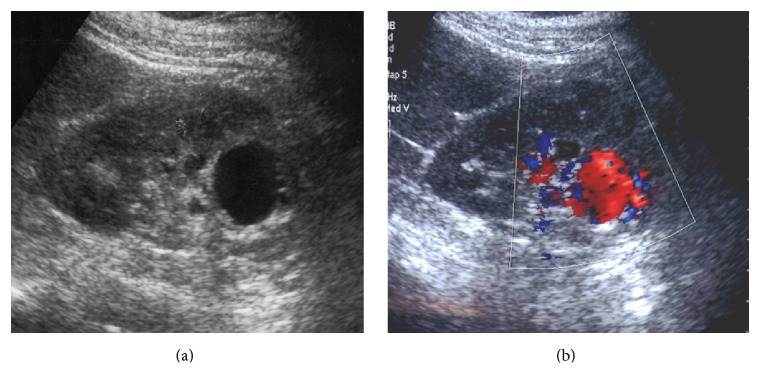
Vascular malformation. Longitudinal gray scale US (a); color Doppler US (b). Gray scale US of the right kidney shows an anechoic renal lesion. At color Doppler vascular flow is depicted within the lesion allowing the diagnosis of a vascular malformation.

Sometimes* vascular anomalies* (such as renal artery aneurysm or arteriovenous fistula) may also mimic a cystic renal lesion on US. When an anechoic lesion is depicted within the renal sinus or in the central part of the kidney it is mandatory to complete the examination with Doppler evaluation (Figure 9). Contrast-enhanced CT or contrast-enhanced MRI can also easily demonstrate the fake cystic nature of the vascular anomaly [7, 9].

### 3.1. Benign Cysts (Categories I and II)

At US examination a simple renal cyst is defined as a rounded, anechoic lesion with a posterior acoustic enhancement, although this last finding is not specific.

At MRI simple cysts are hypointense on T1-weighted sequences and strongly hyperintense at T2-weighted images [7, 9].

According to the Bosniak classification, a benign simple cyst* (category I)* typically shows water attenuation values at CT-scan (<20 HU) without enhancement after i.v. contrast medium administration and a hairline-thin wall and does not contain septa, calcifications, or solid components.

There is no enhancement if the attenuation increases by less than 10 HU [8, 9, 13]. Enhancement is considered unequivocal when the attenuation of the mass increases over 20 HU and ambiguous between 10 and 20 HU (the so-called “pseudoenhancement”) [9].

*Category II* cyst is a benign lesion that may contain a few hairline-thin septa in which perceived (not measurable) enhancement may be appreciated; fine calcification or a short segment of slightly thickened calcification may be present in the wall or septa [7].

**Figure 10:**
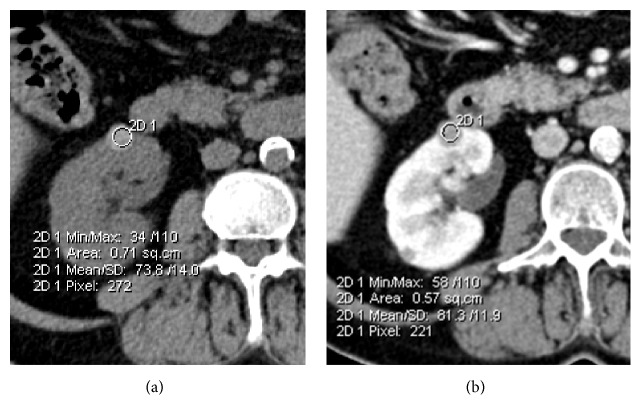
Benign hyperdense renal cyst. Axial CT-scan before (a) and after contrast medium administration (b). The region of interest (ROI) positioned on the small exophytic renal cyst shows high-density content (73 HU) without any significant increase in postcontrastographic study.

This category also includes uniformly high-attenuating lesions smaller than 3 cm, sharply marginated, considered as benign (hemorrhagic or proteinaceous) cysts. It is already known that a renal mass with homogeneous attenuation greater than 70 HU on an unenhanced CT has a greater than 99% probability of being benign (Figure 10) [14].

MRI is helpful in clarifying hemorrhagic cysts found on ultrasound and CT, showing increased signal intensity on T1-weighted and decrease on T2-weighted images, with lack of enhancement after contrast medium injection.

Renal masses included in Bosniak categories I and II are considered benign; therefore they do not require further follow-up or intervention [7].

Although the size is not considered a parameter of Bosniak classification, renal lesions measuring less than 1 cm with simple cysts appearance are statistically likely to represent benign renal cysts. However, the real nature of these masses remains unclear due to their small dimensions.

### 3.2. Low and Medium Risk of Malignancy (Categories IIF and III)

**Figure 11:**
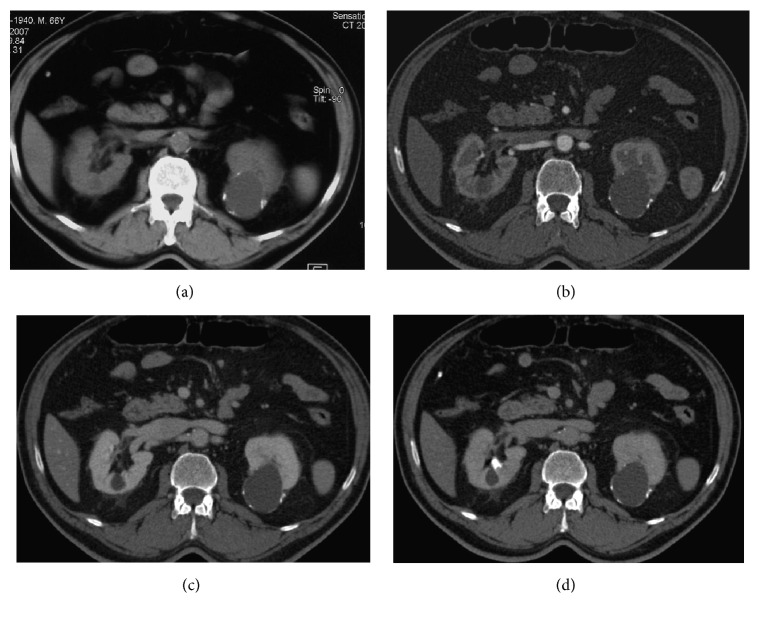
Bosniak category IIF cyst. Axial unenhanced (a) and enhanced CT-scans performed at corticomedullary (b), nephrographic (c), and excretory phase (d). The images show a large hypodense cyst in the left kidney with wall calcifications. A small simple cyst is also visible in the third middle of the right kidney.

*Category IIF* lesions may contain multiple hairline-thin septa. The wall and the septa could be thickened and may contain calcifications, with perceived (not measurable) contrast enhancement [7]. There are no soft tissue enhancing nodules inside (Figure 11).

Nonenhancing high-attenuating renal lesions (<70 HU) that measure more than 3 cm are also included in this category.

These lesions are generally benign but require follow-up imaging (“F” is for follow-up) for morphologic and structural changes, such as development of septa, wall thickening, or new areas of enhancement, suggestive of malignancy [7, 9].

The recommended follow-up consists in a first CT-scan or MRI at 6–12 months, followed by yearly examinations for a minimum of 5 years [9].

*Category III *cysts show thickened irregular or smooth walls or septa manifesting measurable enhancement. This category also includes complicated hemorrhagic or infected cysts, multilocular cystic nephroma, and cystic neoplasms.

**Figure 12:**
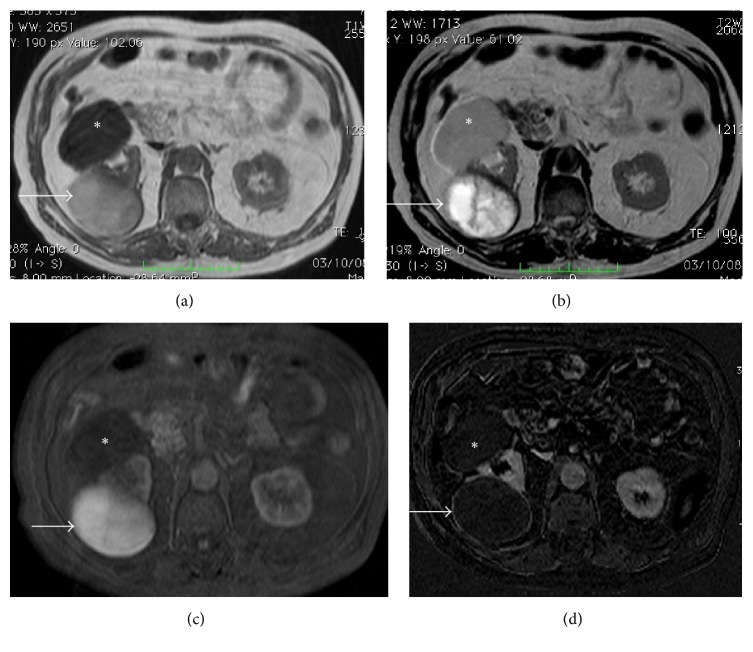
Hemorrhagic cyst. MRI GE T1-weighted (a), TSE T2-weighted (b), contrast-enhanced GE T1-weighted fat-sat (c), and subtracted postcontrastographic (d) image. A large cystic lesion is detectable on the right kidney (arrows), characterized by mild hyperintensity on GE T1-weighted images and inhomogeneous hyperintensity on T2-weighted and on contrast-enhanced GE T1-weighted fat-sat images. On subtracted postcontrastographic image (d) the lesion shows a regular thin wall with mild enhancement. The final histological diagnosis was hemorrhagic cyst. Note also an anterior huge simple cyst (asterisk) in the same kidney.

These masses are considered indeterminate, because a malignant neoplasm cannot be excluded. Therefore, a histologic diagnosis and, in many cases, also surgical intervention are required (Figure 12) [7].

**Figure 13:**
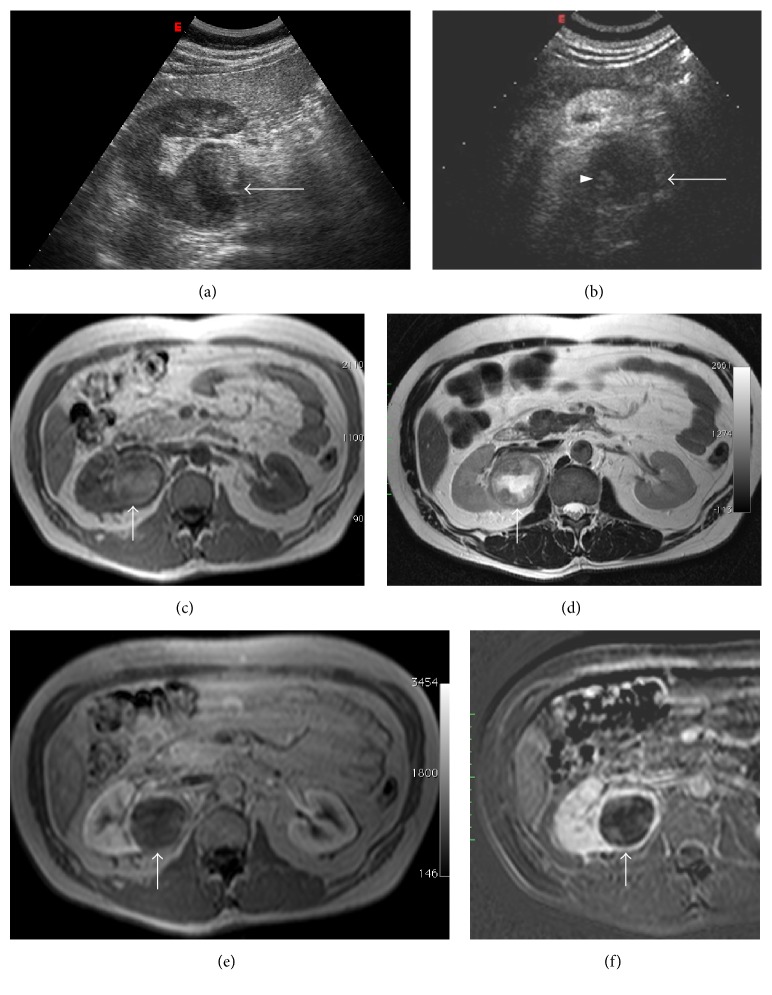
Cystic neoplasm (Bosniak category III). US examination shows a large inhomogeneous hypoechoic mass (arrow) in the third middle of the right kidney (a). At CEUS, enhancement of intralesional septa and nodulations (arrowhead) is also detectable (b). MRI in the same patient (c–f). The mass (arrow) is characterized by an inhomogeneous mild hyperintensity on axial GE T1-weighted image (c) and a central area of hyperintensity on axial T2-weighted TSE image (d). Axial contrast-enhanced GE T1-weighted fat-sat image (e) and subtracted image (f): enhancement of the small solid peripheral component is better depicted on the subtracted image, with similar CEUS appearance.

Differentiation between category IIF and category III can be challenging, due to the variable appearance and the radiologists' experience, and often require more than one imaging modality (Figure 13).

However, it is always extremely important to define in order to establish the correct management.

Contrary to the common opinion, it should be considered that a small percentage of category III masses can be benign (the range of malignancy is between 31% and 100%). Despite this consideration, surgery is the treatment of choice in order to avoid a misdiagnosis.

### 3.3. High Risk of Malignancy (Category IV)

*Category IV* includes cystic masses with the same characteristics of category III with a distinct enhancing of soft tissue components independent of the wall or septa.

**Figure 14:**
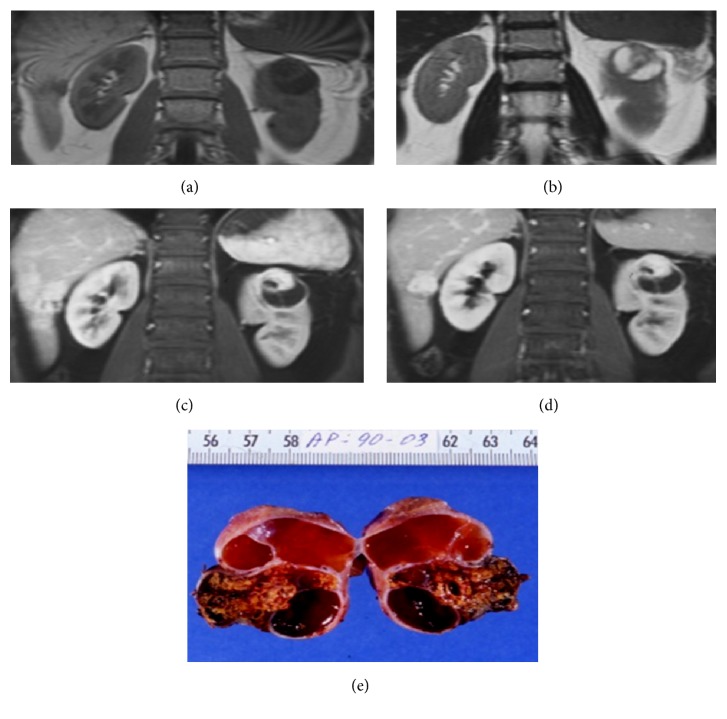
Bosniak IV type cystic lesion. Coronal GE T1-weighted image (a); coronal TSE T2-weighted image (b); contrast-enhanced coronal GE-T1 weighted image in corticomedullary (c) and nephrographic (d) phases. The cystic lesion with huge high enhanced solid parietal nodule is well depicted. Note also the good anatomoradiological correlation between the MR examination and histologic specimen (d). Note also a small liver hemangioma.

These lesions are clearly malignant and need to be surgically removed (Figure 14) [7, 9].

## 4. Solid Renal Masses

Solid renal masses are structurally characterized by little or no fluid components usually containing predominantly enhancing tissue. Although a mass-like renal abnormality with these appearances could be a consequence of infarction and infection as well as trauma, clinical history is usually indicative of these illnesses.

Depending on the final treatment, solid renal masses can be distinguished in surgical and nonsurgical lesions.

Lymphoma and renal metastases were excluded from this topic because of their less frequent discovery as incidental lesions, due to the clinical history of the patient (e.g., extrarenal primary malignancy) and the frequent involvement of other anatomical districts.

### 4.1. Nonsurgical Lesions

*Renal angiomyolipomas* (AMLs) are the most common benign renal tumors, now considered among the family of perivascular epithelioid cell tumors (PEComa) and divided into two histological categories: triphasic AMLs and monotypic epithelioid AMLs [15].

While the latter ones represent an extremely rare and potentially malignant type, containing few or no fat cells, triphasic AMLs are the most common, with variable amount of vascular, muscular, and adipose components, and further categorized into classic and fat-poor lesions.

The frequent hyperechogenicity of AML at US is not specific and requires further evaluations to rule out a RCC. Although CEUS can potentially add diagnostic value, frequently showing a peripheral enhancement pattern, cross-sectional imaging is needed [10, 16].

**Figure 15:**
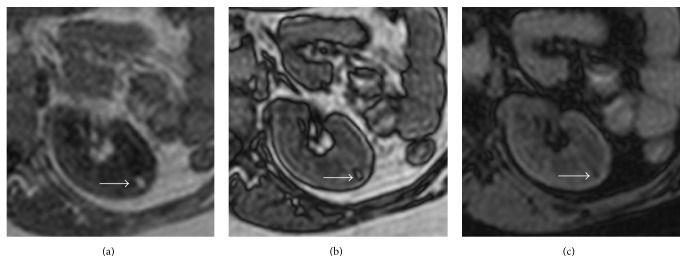
Small left kidney typical AML. Axial GE T1-weighted IP image (a); axial GE T1-weighted OP image (b); axial GE T1-weighted fat-sat image. A small renal AML with typical appearance (arrow): high signal intensity on T1-weighted IP image, “India-ink” artifact on T1-weighted OP image, and loss of signal intensity on T1-weighted fat-sat image.

In fact detection of macroscopic fat in a renal lesion is a specific finding of* Classic AMLs*, which are typically hypodense at CT-scan (<10 HU), hyperintense on T1- and T2-weighted sequences at MRI, and with loss of signal following frequency-selective fat-saturation technique; other typical MRI features include high signal intensity on T1-weighted GE in-phase (IP) and opposed-phase sequences (OP) with signal dropout on opposed-phase at the interface of the lesion with the normal parenchyma (“India-ink” artifact) (Figure 15) and high signal intensity on fat-only reconstruction from Dixon-based acquisitions.

Fat-poor AMLs (5% of all AMLs) without detectable fat on imaging cannot be differentiated from other renal masses, due to the lack of a typical appearance.

In particular, depending on fat-cell distribution and amount, fat-poor AMLs can appear as hyperattenuating (>45 HU) or isoattenuating (−10 to 45 HU) at CT-scan [15].

Moreover, MRI features overlap with RCCs; indeed, the typical T2-weighted low signal intensity of fat-poor AMLs can almost exclude a clear cell RCC but not a papillary RCC (although a small proportion of clear cell RCCs and chromophobe RCCs also had low signal intensity on T2-weighted images).

Furthermore, a signal loss in opposed-phase images cannot be used to accurately distinguish minimal fat AMLs from clear cell RCCs, which may present intracytoplasmic lipid-containing vacuoles.

Additionally, it should be noticed that the presence of necrosis virtually excludes the diagnosis of AML [16].

Other considerations were made about contrast-enhancement pattern and DWI in order to differentiate AMLs from RCCs, but the literature's data are not always univocal.

According to Sasiwimonphan et al. [17] the so-called arterial/delayed enhancement ratio (defined as the difference in signal intensity between arterial and precontrast phase divided by the difference between delayed and precontrast phase) can be helpful in differentiating poor fat AMLs from RCCs with values greater than 1.5 favoring the first [15]. However, more recently, Hakim et al. demonstrated that the contrast-enhancement pattern cannot be reliable due to overlap with the clear cell RCC enhancement [18].

Even DWI showed ambiguous results in differentiating AMLs from malignant masses.

Indeed, though some authors described a possible differential diagnosis between AMLs and RCCs depending on ADC map [19], up to now these data are not sufficiently reliable due to the great variability of the *b* values used, the MR field strength of the scanner, and even between individual readers picking the region of interest (ROI) [15].

*Oncocytoma* is the second benign renal tumor (3–9% of all primary renal neoplasms), hypo- or isoechoic solid mass at US, with homogeneous CT-attenuation values if small (<3 cm) and heterogeneous if large (>3 cm) and a T1-hypointensity and a T2-hyperintensity at MRI.

More typical features of oncocytoma, when present, are the central scar and the arterial spoke-wheel pattern of enhancement; moreover, at CT, in small oncocytomas (<4 cm) the “segmental inversion enhancement pattern” was recently described that is based on the presence of two distinct regions of enhancement in the corticomedullary phase (30–40 s) in which its degree reverses in the nephrographic phase (120–180 s) [9, 13, 20].

Unfortunately, all the imaging findings described up to now are not specific for oncocytoma and the final diagnosis is generally reached with biopsy [13].

However, recently, some authors evaluated if DWI can play a role in distinguishing oncocytoma from malignant lesions, reporting a significant difference with higher ADC values for the first ones [15, 16, 21, 22].

### 4.2. Surgical Lesions

RCC is the eighth most common tumor in the adulthood (2-3% of adult cancers) and the first tumor in the urinary tract (90%) [9, 23].

The most common histological subtypes are clear cell (cc-RCC, 75%), papillary (p-RCC, 10–15%), and chromophobe (ch-RCC, 5%) RCCs, with a better outcome for the last two [16, 24].

At US, RCCs are usually depicted as hypoechoic or isoechoic masses; Doppler and CEUS may be useful in depicting renal vein or inferior vena cava thrombosis [13] and in evaluating the vascularization of the mass but did not show a sufficient accuracy in differentiating the histological subtypes of RCCs [16, 25–27].

On noncontrast CT, RCCs are usually characterized by a soft tissue attenuation, except for larger lesions that can show heterogeneous content. Enhanced CT may be helpful in differentiating the tumor subtypes, magnifying the histological characteristics. Indeed, due to its rich vascular network, clear cell RCC manifests stronger enhancement in both the corticomedullary and excretory phases, while papillary and chromophobe tumors, which are less vascularized, tend to manifest a lower, homogeneous, and more peripheral enhancement.

**Figure 16:**
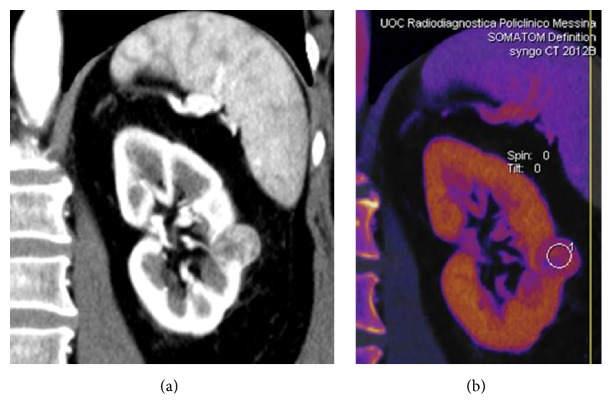
Small cc-RCC. Coronal reformatted DE arterial-phase CT image (a); coronal color-coded iodine overlay image (b). High enhanced solid renal mass with an high iodine content (2.4 mgI on ROI).

Moreover, on dual-energy CT, the determination of iodine content on color-coded iodine overlay dual-energy images can allow an earlier recognition of clear cell histotype, which is the most aggressive RCC, with a significant improvement of patient's outcome (Figure 16) [28–31].

**Figure 17:**
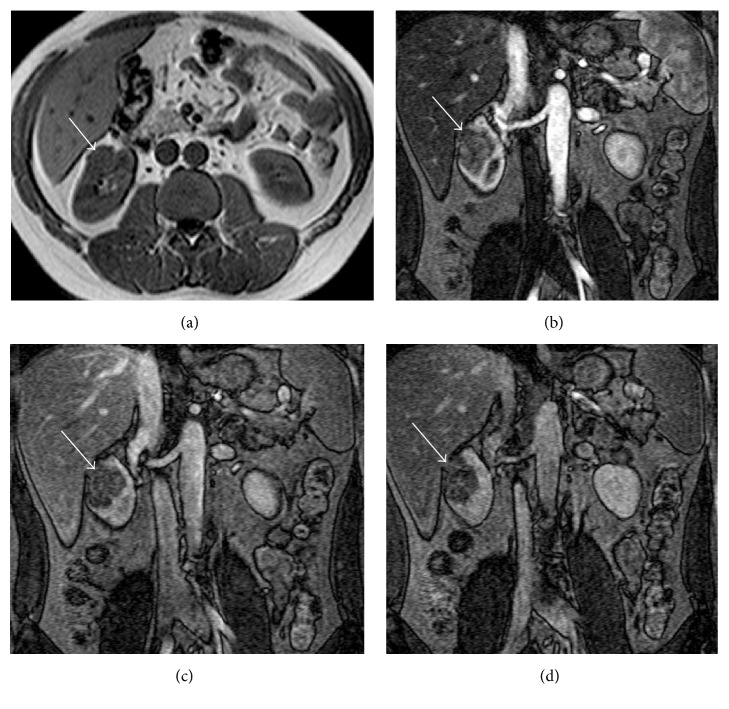
P-RCC. Axial GE T1-weighted image (a); coronal GE T1-weighted dynamic contrast-enhanced MR scans (b–d). Exophytic isointense nodule (arrow) in the right kidney with poor enhancement in multiphasic study.

At MRI all RCCs are fundamentally hyperintense at T2-weighted sequences except for the papillary subtype, because of its hypovascularity (Figure 17). T1 signal intensity is always variable, depending on the presence of intralesional degeneration areas (hemorrhagic, cystic, or necrotic). After contrast medium administration, MRI shows the same enhancement patterns described for contrast-enhanced CT and can be useful in depicting renal vein and inferior vena cava involvement.

Although several recent studies have evaluated the use of DWI in RCCs, showing higher ADC values in cc-RCCs than papillary and chromophobic types, up to now it cannot be used in distinguishing among the different histotypes [32, 33].

However, imaging differentiation of the histological subtypes of RCCs may be unnecessary, considering that the characterization is reached by biopsy and the treatment is anyway surgical intervention.

## 5. Conclusions

Despite the substantial advances in the imaging-based diagnosis of the last decades, the characterization of incidental renal lesions still remains one of the most challenging topics for the radiologist.

Although cross-sectional imaging can confidently distinguish almost all large masses, the major critic point concerns the correct stratification of complex cysts and the characterization of small solid lesions.

**Figure 18:**
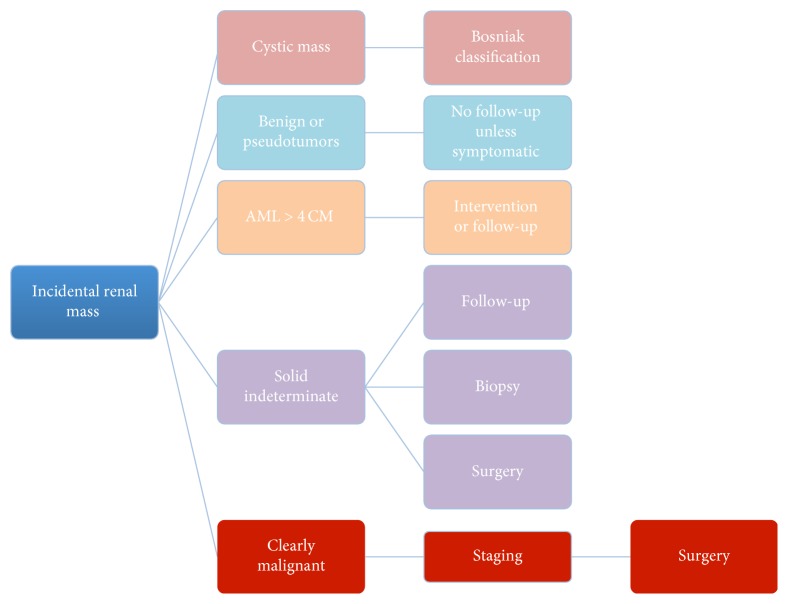
Management diagram.

Based on the probability of malignancy, active surveillance or biopsy can be suggested in order to avoid useless and more invasive treatments (like percutaneous ablation or surgical intervention) (Tables 1 and 2) (Figure 18).

In conclusion, the decision about the management of incidental renal lesions cannot simply result from depicting of radiological findings but should be focused on the patient, considering his anamnestic data (comorbidities, life expectancy) and clinical history.

## Figures and Tables

**Table 1:** Cystic renal lesions.

Type	Definition	US	CT	MRI	Percentage of malignancy	Management
Benign	*Bosniak class I* simple cyst not containing septa or calcification; no enhancement	(i) Anechoic (ii) Thin, smooth walls (iii) Posterior acoustic enhancement	(i) Near water density (<10 HU) (ii) Homogeneous density (iii) No enhancement	(i) Sharp, well-defined walls (ii) Signal characteristics of water (iii) No enhancement	~0%	No intervention
	*Bosniak class II* cystic lesion containing thin septa or fine calcifications; no enhancement	(i) Hypoechoic (ii) Iso- or hyperechoic < 3 cm	(i) Hypodense (ii) Hyperdense < 3 cm	(i) T1 hypointense T2 hyperintense (ii) T1 hyperintense T2 hypointense (<3 cm)		

Minimally complex	*Bosniak class IIF* (i) Multiple septa (ii) Perceived (not measurable) enhancement (iii) Calcifications	(i) Hypoechoic (ii) Iso- or hyperechoic > 3 cm	(i) Hypodense (ii) Hyperdense > 3 cm	(i) T1 hypointense T2 hyperintense (ii) T1 hyperintense T2 hypointense (>3 cm)	5%	Follow-up

Indeterminate and malignant	*Bosniak class III* (i) Thickened irregular wall or septa (ii) Thick calcifications (iii) Measurable enhancement				55%	Histologic diagnosis and eventual surgical removal
	*Bosniak class IV* (i) Bosniak class III parameters and solid nodular components				100%	Surgical removal

**Table 2:** Nodular solid lesions (“ball” type).

Type	Common diagnosis	CT	MRI
Benign	AML	(i) Macroscopic fat tissue at unenhanced CT (ii) No calcifications	(i) T1 hyperintense (ii) T2 hypointense (iii) India-ink artifact at the interface between the mass and the renal parenchyma (intracellular fat)

Indeterminate	Oncocytoma	(i) Homogeneous intravascular pattern (ii) Central scar (iii) Segmental inversion enhancement pattern	(i) Not specific: usually T1-hypointensity and T2-hyperintensity (ii) Scar: T2 hyperintense (iii) High signal on ADC

Malignant	RCC	(i) Hypervascularity (cc-RCC) (ii) Hypovascularity (p-RCC, ch-RCC) (iii) Homogeneous peripheral enhancement (p-RCC) (iv) Moderate enhancement (ch-RCC)	(i) T1 isointense and T2 hyperintense (cc-RCC) (ii) T1 hyperintense and T2 hypointense (p-RCC and ch-RCC)
